# Early vascular aging determined by brachial-ankle pulse wave velocity and its impact on ischemic stroke outcome: a retrospective observational study

**DOI:** 10.1038/s41598-024-62847-w

**Published:** 2024-06-13

**Authors:** Minho Han, Jaeseob Yun, Kwang Hyun Kim, Jae Wook Jung, Young Dae Kim, JoonNyung Heo, Eunjeong Park, Hyo Suk Nam

**Affiliations:** 1 Department of Neurology, Yonsei University College of Medicine, 50-1 Yonsei-Ro, Seodaemoon-Gu, Seoul, 03722 South Korea; 2 Integrative Research Center for Cerebrovascular and Cardiovascular Diseases, Yonsei University College of Medicine, Seoul, South Korea

**Keywords:** Vascular aging, Arterial stiffness, Stroke, Prognosis, Biomarkers, Diseases, Medical research, Neurology, Risk factors

## Abstract

Vascular aging phenotype may be useful in predicting stroke prognosis. In the present study, the relationship between vascular aging phenotypes and outcomes after acute ischemic stroke was investigated. The study included consecutive patients with acute ischemic stroke who had brachial-ankle pulse wave velocity (baPWV) measured to assess vascular aging phenotype. The 2.5th and 97.5th percentile age-specific baPWVs were used as cutoffs to define supernormal vascular aging (SUPERNOVA) and early vascular aging (EVA), respectively, and the remainder was considered normal vascular aging (NVA). A total of 2738 patients were enrolled and followed for a median of 38.1 months. The mean age was 67.02 years and 1633 were male. EVA was 67, NVA was 2605, and SUPERNOVA was 66. Compared with NVA, multivariable logistic regression showed EVA was associated with poor functional outcome (modified Rankin Scale ≥ 3) at 3 months (odds ratio 2.083, 95% confidence interval 1.147‒3.783). Multivariable Cox regression showed EVA was associated with all-cause mortality (hazard ratio 2.320, 95% confidence interval 1.283‒4.197). EVA was associated with poor functional outcome and all-cause mortality after acute ischemic stroke, especially when diabetes or atrial fibrillation coexisted. These findings indicate the vascular aging phenotype, notably EVA, can aid in identifying high-risk stroke patients.

## Introduction

Vascular aging is a process that begins in utero and continues until death. This natural process is accelerated by pro-aging factors and leads to the early onset of cardiovascular disease^1^. Early vascular aging (EVA) identifies individuals with accelerated aging due to genetics, environmental interactions, or arterial damage leading to medial layer changes. EVA can be diagnosed in subjects who present with abnormally high arterial stiffness for their age and sex, which is easily measured by non-invasive methods such as brachial-ankle pulse wave velocity (baPWV)^2^. Understanding the pathophysiology of EVA may improve outcomes and survival after cardiovascular disease^3^.

Due to the relationship between vascular aging and stiffness, the extent of arterial stiffness can indicate specific vascular aging phenotypes such as supernormal vascular aging (SUPERNOVA) as well as EVA^4^. Subjects with EVA have arteries that are much stiffer than individuals without EVA, and vice versa for SUPERNOVA^5^. Arterial stiffness is a well-established predictor of cardiovascular disease^6^. In particular, arterial stiffness is considered a strong determinant of poor outcomes in patients with acute ischemic stroke^7^. However, the relationship between the phenotype of EVA or SUPERNOVA and stroke prognosis has not been elucidated. In the present study, the vascular aging phenotypes associated with short- and long-term outcomes in patients with acute ischemic stroke were investigated.

## Results

### Demographics

Among 4136 patients with acute ischemic stroke, subjects who did not undergo baPWV measurements (n = 1111) were excluded. After further excluding patients with an ankle-brachial index ≤ 0.9 (n = 287) for the reliability of baPWV^8^, a total of 2738 patients were finally included (Supplementary Fig. 1). The mean age was 67.02 ± 13.53 years and 1633 (59.6%) were male. The median National Institutes of Health Stroke Scale (NIHSS) score at admission was 2.0 (interquartile range 1.0‒4.0). EVA, normal vascular aging (NVA), and SUPERNOVA accounted for 2.5%, 95.0%, and 2.5% of patients, respectively. The baPWV was highest in EVA patients (37.87 ± 12.52 m/s), followed by NVA (19.70 ± 4.71 m/s) and SUPERNOVA (12.23 ± 1.56 m/s; p < 0.001).

**Figure1:**
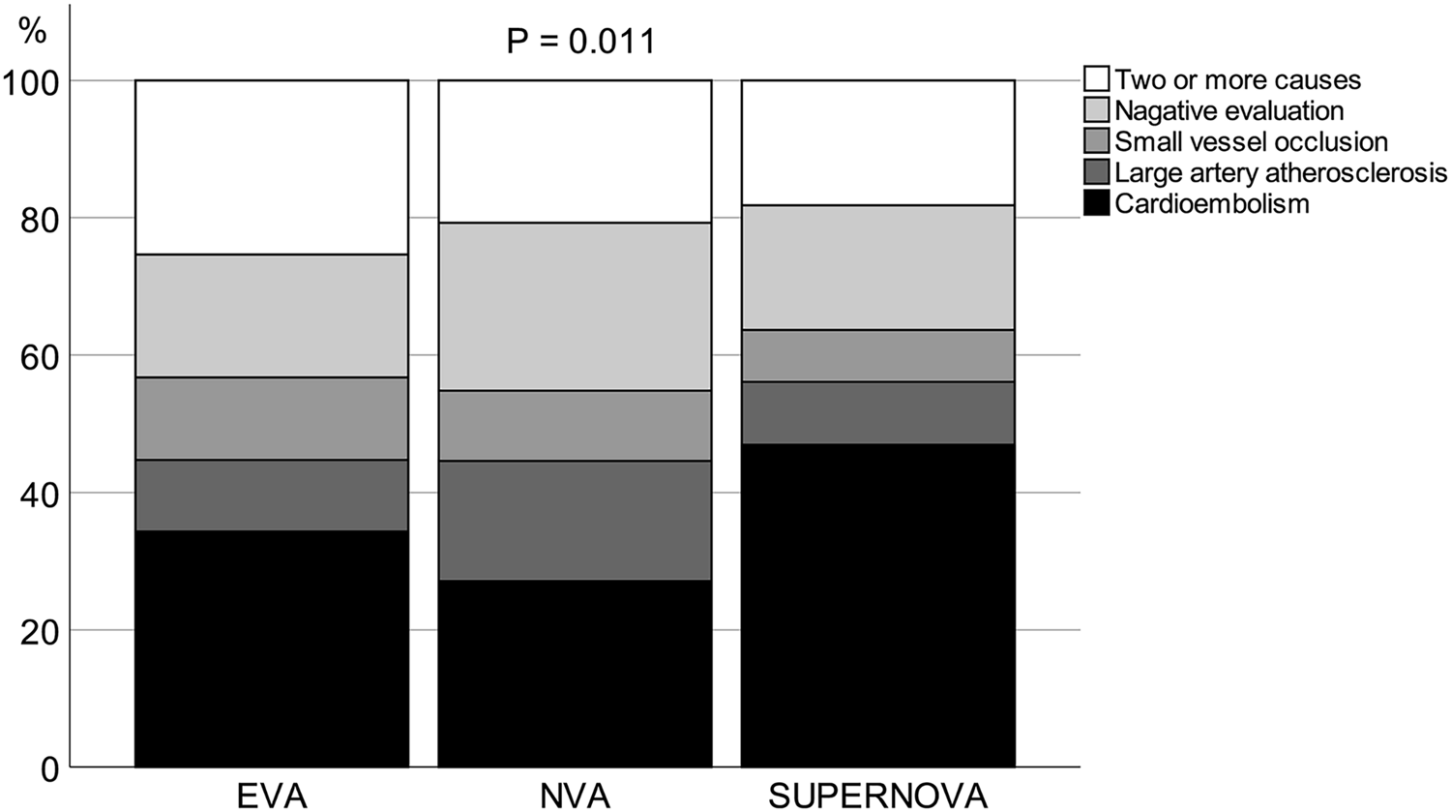
Distribution of stroke subtypes based on vascular aging. *EVA* early vascular aging, *NVA* normal vascular aging, *SUPERNOVA* supernormal vascular aging. P value was obtained by the Chi-square test.

Age and sex did not differ among EVA, NVA, and SUPERNOVA subjects. The prevalence of hypertension was highest in EVA patients (85.1%), followed by NVA (74.1%) and SUPERNOVA (51.5%; p < 0.001). Diabetes was most prevalent in EVA subjects (53.7%), followed by NVA (30.7%) and SUPERNOVA (19.7%; p < 0.001). Atrial fibrillation was also most frequent in EVA patients (35.8%), followed by SUPERNOVA (31.8%) and NVA (19.7%; p < 0.001) (Table 1). Among stroke subtypes, cardioembolism was most common in patients with SUPERNOVA (47.0%), followed by EVA (34.3%) and NVA (27.1%; p = 0.001). Large artery atherosclerosis and small vessel occlusion had similar prevalence among the vascular aging phenotypes (Fig. 1 and Table 1).

**Table 1:** Patient demographic and clinical characteristics.

	EVA (n = 67)	NVA (n = 2605)	SUPERNOVA (n = 66)	Total (n = 2738)	*p*-value
Age, years	68.33 ± 14.25	67.02 ± 13.51	65.88 ± 13.76	67.02 ± 13.53	0.578
Sex (men)	33 (49.3)	1565 (60.1)	35 (53.0)	1633 (59.6)	0.110
Body mass index, kg/m^2^	23.6 [21.3, 24.9]	23.9 [22.0, 25.8]	23.1 [20.9, 25.8]	23.9 [21.9, 25.8]	0.066
NIHSS at admission	3.0 [1.0, 5.0]	2.0 [1.0, 4.0]	2.0 [1.0, 6.0]	2.0 [1.0, 4.0]	0.333
Risk factors					
Hypertension	57 (85.1)	1930 (74.1)	34 (51.5)	2021 (73.8)	< 0.001
Diabetes	36 (53.7)	801 (30.7)	13 (19.7)	850 (31.0)	< 0.001
Dyslipidemia	14 (20.9)	510 (19.6)	12 (18.2)	536 (19.6)	0.926
Current smoking	10 (14.9)	580 (22.3)	11 (16.7)	601 (22.0)	0.195
Atrial fibrillation	24 (35.8)	513 (19.7)	21 (31.8)	558 (20.4)	< 0.001
Coronary artery disease	23 (34.3)	767 (29.4)	17 (25.8)	807 (29.5)	0.549
Previous stroke	15 (22.4)	440 (16.9)	12 (18.2)	467 (17.1)	0.483
Blood tests					
Total cholesterol, mg/dL	173.5 [148.8, 210.0]	171.0 [142.0, 199.0]	164.5 [134.0, 193.3]	171.0 [142.0, 199.0]	0.337
HDL-C, mg/dL	44.0 [37.0, 52.3]	42.0 [36.0, 51.0]	43.0 [35.0, 47.8]	42.0 [36.0, 51.0]	0.506
LDL-C, mg/dL	108.5 [81.5, 142.0]	101.0 [77.0, 125.0]	105.5 [73.3, 129.0]	101.0 [77.0, 126.0]	0.258
Triglyceride, mg/dL	103.0 [74.8, 140.5]	102.0 [75.0, 144.0]	90.5 [63.3, 120.8]	102.0 [75.0, 143.0]	0.059
Stroke subtypes					
Small vessel occlusion (SVO)	8 (11.9)	265 (10.2)	5 (7.6)	278 (10.2)	0.669^a^
Large artery atherosclerosis (LAA)	7 (10.4)	456 (17.5)	6 (9.1)	469 (17.1)	0.068^a^
Cardioembolism (CE)	23 (34.3)	706 (27.1)	31 (47.0)	760 (27.8)	0.001^a^
Undetermined causes (UC)	29 (43.3)	1178 (45.2)	24 (36.4)	1231 (45.0)	0.347^a^
UC, negative evaluation	12 (17.9)	638 (24.5)	12 (18.2)	662 (24.2)	0.238^a^
UC, ≥ 2 causes identified	17 (25.4)	540 (20.7)	12 (18.2)	569 (20.8)	0.567^a^
≥ 2 causes, LAA + CE	12 (17.9)	251 (9.6)	5 (7.6)	268 (9.8)	0.066^a^
≥ 2 causes, LAA + SVO	2 (3.0)	143 (5.5)	4 (6.1)	149 (5.4)	0.670^a^
≥ 2 causes, LAA + CE + SVO	1 (1.5)	17 (0.7)	1 (1.5)	19 (0.7)	0.235^a^
≥ 2 causes, CE + SVO	2 (3.0)	129 (5.0)	2 (3.0)	133 (4.9)	0.626^a^

*EVA* early vascular ageing, *HDL-C* high-density lipoprotein cholesterol, *LDL-C* low-density lipoprotein cholesterol, *NIHSS* National Institutes of Health Stroke Scale, *NVA* normal vascular ageing, *SUPERNOVA* supernormal vascular ageing.

^a^p-values obtained by comparing groups with and without a given stroke subtype.

### Association between vascular aging and functional outcome at 3 months

Univariable logistic regression analysis showed poor functional outcome was significantly associated with EVA, along with age, sex, body mass index, initial stroke severity, hypertension, diabetes, smoking, atrial fibrillation, coronary artery disease, previous stroke, triglyceride, and stroke subtypes (all p-values < 0.05). Multivariable analysis showed EVA was independently associated with poor functional outcome at 3 months (odds ratio [OR] 2.083, 95% confidence interval [CI] 1.147‒3.783, p = 0.016); however, SUPERNOVA was not associated with functional outcome (OR 0.516, 95% CI 0.229‒1.162, p = 0.110; Table 2).

**Table 2:** Logistic regression analysis of poor functional outcome at 3 months.

	Univariable		Multivariable^a^	
	OR (95% CI)	*p*-value	OR (95% CI)	*p*-value
Age, years	1.045 (1.037‒1.054)	< 0.001	1.036 (1.027‒1.047)	< 0.001
Sex (men)	0.650 (0.539‒0.784)	< 0.001	0.767 (0.608‒0.968)	0.025
Body mass index, kg/m^2^	0.945 (0.918‒0.973)	< 0.001	0.974 (0.942‒1.008)	0.137
NIHSS at admission	1.218 (1.192‒1.245)	< 0.001	1.219 (1.190‒1.248)	< 0.001
Hypertension	1.361 (1.089‒1.699)	0.007	1.084 (0.826‒1.421)	0.561
Diabetes	1.316 (1.082‒1.601)	0.006	1.258 (0.999‒1.584)	0.051
Dyslipidemia	1.087 (0.862‒1.369)	0.481		
Current smoking	0.731 (0.576‒0.927)	0.010	1.153 (0.855‒1.557)	0.351
Atrial fibrillation	1.847 (1.493‒2.285)	< 0.001	0.767 (0.550‒1.070)	0.119
Coronary artery disease	0.808 (0.654‒0.996)	0.046	0.837 (0.659‒1.063)	0.144
Previous stroke	1.534 (1.218‒1.932)	< 0.001	1.325 (1.015‒1.731)	0.039
Total cholesterol, mg/dL	0.998 (0.996‒1.000)	0.053		
HDL-C, mg/dL	0.999 (0.991‒1.008)	0.870		
LDL-C, mg/dL	0.999 (0.996‒1.001)	0.338		
Triglyceride, mg/dL	0.998 (0.997‒0.999)	0.004	1.000 (0.999‒1.001)	0.828
Stroke subtypes				
Small vessel occlusion	Reference		Reference	
Large artery atherosclerosis	1.859 (1.240‒2.786)	0.003	1.415 (0.916‒2.185)	0.117
Cardioembolism	1.924 (1.314‒2.816)	0.001	1.140 (0.722‒1.798)	0.574
Undetermined causes	1.485 (1.025‒2.152)	0.036	1.176 (0.792‒1.746)	0.422
Vascular aging				
NVA	Reference		Reference	
SUPERNOVA	0.707 (0.358‒1.395)	0.318	0.516 (0.229‒1.162)	0.110
EVA	2.118 (1.265‒3.546)	0.004	2.083 (1.147‒3.783)	0.016

Poor functional outcome is defined as the modified Rankin Scale score ≥ 3 at 3 months. *CE* cardioembolism, *CI* confidence interval, *EVA* early vascular ageing, *HDL-C* high-density lipoprotein cholesterol, *LAA* large artery atherosclerosis, *LDL-C* low-density lipoprotein cholesterol, *NIHSS* National Institutes of Health Stroke Scale, *NVA* normal vascular ageing, *OR* odds ratio, *SUPERNOVA* supernormal vascular ageing, *SVO* small vessel occlusion.

^a^Adjusted for age, sex, body mass index, NIHSS score at admission, hypertension, diabetes, current smoking, atrial fibrillation, coronary artery disease, previous stroke, triglyceride, and stroke subtypes.

### Association between vascular aging and long-term outcomes

**Figure 2:**
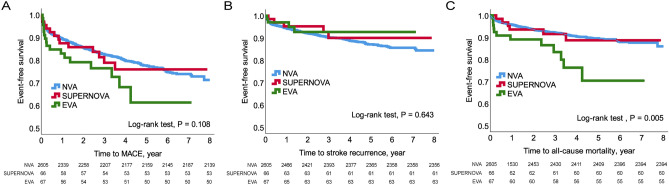
Kaplan–Meier plots according to vascular aging phenotypes. Survival curves for MACE (**A**), stroke recurrence (**B**), and all-cause mortality (**C**). *EVA* early vascular aging, *MACE* major adverse cardiovascular event, *NVA* normal vascular aging, *SUPERNOVA* supernormal vascular aging.

Patients were followed for a median of 38.1 months (interquartile range, 19.8–61.8 months). A total of 496 patients (18.1%) experienced a major adverse cardiovascular event (MACE) during the study period; 258 (9.4%), stroke recurrence; 9 (0.3%), acute coronary syndrome; and 229 (8.4%), all-cause mortality. The Kaplan–Meier curve analysis showed EVA was significantly associated with all-cause mortality (p = 0.005); however, SUPERNOVA did not differ from NVA (Fig. 2). After adjusting for potential confounders (Supplementary Table 1), Cox regression analysis showed all-cause mortality was independently associated with EVA (hazard ratio [HR] 2.320, 95% CI 1.283‒4.197, p = 0.005) but not with SUPERNOVA (HR 1.206, 95% CI 0.534‒2.725, p = 0.652). In the model adjusted for atherosclerotic cardiovascular disease (ASCVD) risk factors, EVA remained an independent determinant of all-cause mortality. However, EVA and SUPERNOVA did not show an independent association with MACE and stroke recurrence (Table 3).

**Table 3:** Multivariable Cox regression analysis of long-term outcomes.

	Model 1		Model 2	
	HR (95% CI)	*p-*value	HR (95% CI)	*p-*value
MACE				
NVA	Reference		Reference	
SUPERNOVA	1.054 (0.592‒1.875)	0.858	1.077 (0.606‒1.914)	0.801
EVA	1.388 (0.837‒2.301)	0.204	1.526 (0.924‒2.520)	0.099
Stroke recurrence				
NVA	Reference		Reference	
SUPERNOVA	0.842 (0.346‒2.048)	0.704	0.862 (0.354‒2.095)	0.742
EVA	0.609 (0.226‒1.645)	0.328	0.482 (0.154‒1.510)	0.211
All-cause mortality				
NVA	Reference		Reference	
SUPERNOVA	1.206 (0.534‒2.725)	0.652	1.181 (0.523‒2.668)	0.688
EVA	2.320 (1.283‒4.197)	0.005	2.708 (1.505‒4.873)	0.001

*CI* confidence interval, *EVA* early vascular ageing, *HR* hazard ratio, *MACE* major adverse cardiovascular event, *NIHSS* National Institutes of Health Stroke Scale, *NVA* normal vascular ageing, *SUPERNOVA* supernormal vascular ageing.

Model 1: adjusted for age, sex, body mass index, NIHSS at admission, and variables with p < 0.05 in univariable analysis.

Model 2: adjusted for the atherosclerotic cardiovascular disease risk factors (age, sex, hypertension, diabetes, current smoking, total cholesterol, and high-density lipoprotein cholesterol).

### Subgroup analysis of poor outcomes

**Figure 3:**
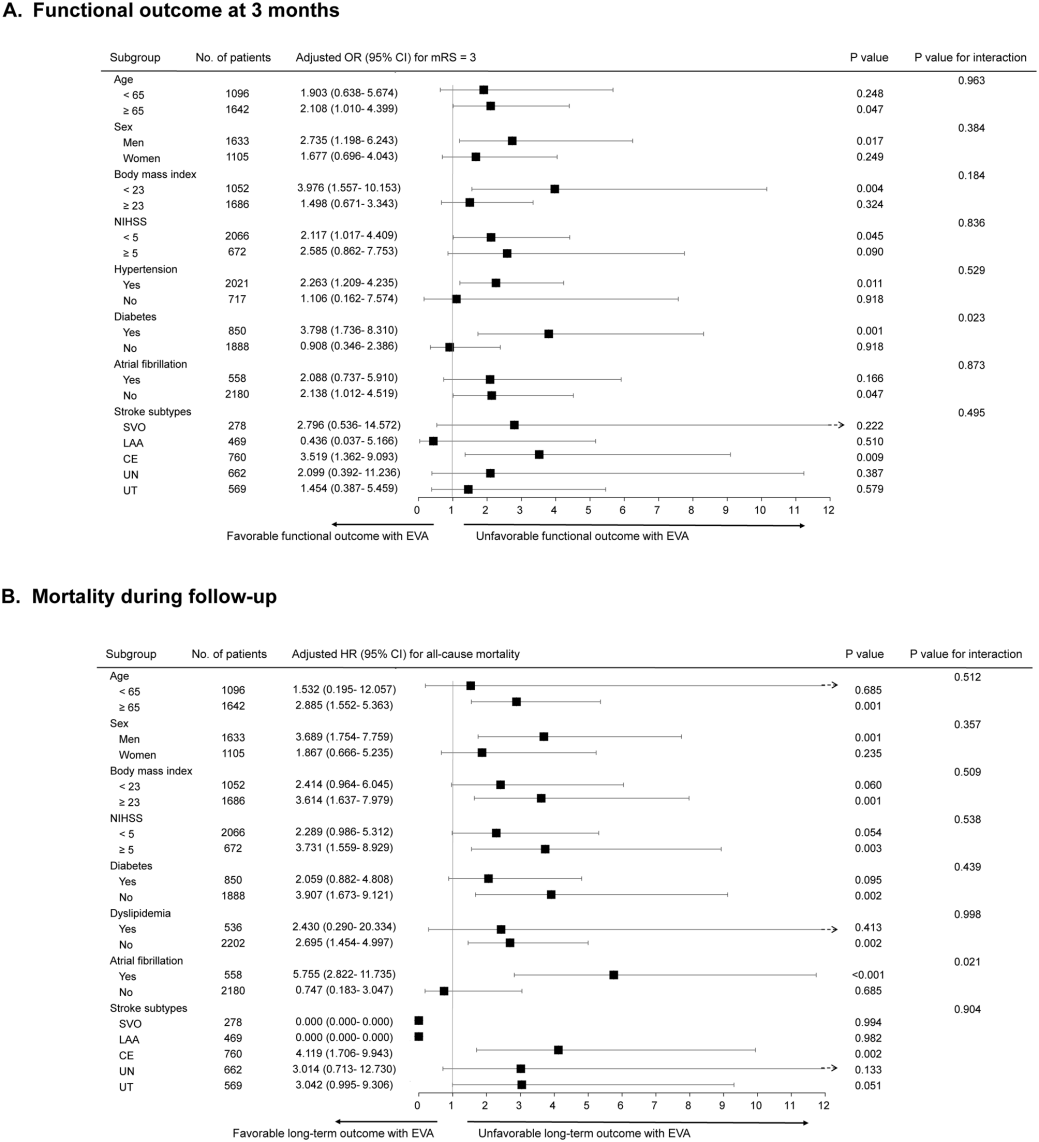
Subgroup analysis based on patient characteristics. Multivariable logistic regression and multivariable Cox regression were utilized for functional outcome (**A**) and mortality (**B**), respectively. *CE* cardioembolism, *CI* confidence interval, *EVA* early vascular aging, *HR* hazard ratio, *LAA* large artery atherosclerosis, *NIHSS* National Institutes of Health Stroke Scale, *OR* odds ratio, small vessel occlusion, *UN* negative evaluation, *UT* two or more causes.

Multivariable logistic or Cox regression was utilized for subgroup analysis. In all strata, the effect of EVA tended to be associated with poor functional outcome and frequent all-cause mortality. However, a significant interaction was observed in patients with diabetes or atrial fibrillation. The presence of EVA was significantly associated with poor functional outcome in patients with diabetes (OR 3.798, 95% CI 1.736‒8.310, p = 0.001) but not in subjects without (p-value for interaction = 0.023; Fig. 3A). In addition, EVA was significantly associated with all-cause mortality in patients with atrial fibrillation (OR 5.755, 95% CI 2.822‒11.735, p < 0.001) but not in subjects without (p-value for interaction = 0. 021; Fig. 3B).

## Discussion

In this study, the presence of EVA was independently associated with poor functional outcome at 3 months and all-cause mortality after acute ischemic stroke. The unfavorable effect of EVA was more noticeable on functional outcome in patients with diabetes and on mortality in those with atrial fibrillation. In contrast, the prognosis of SUPERNOVA was similar to NVA in patients with acute ischemic stroke.

The hallmark of vascular aging is arterial stiffness, which can be readily assessed by measuring baPWV. This measurement serves as a valuable tool to evaluate the present state of arterial health influenced by age, risk factors, and natural susceptibility^9^. In addition, baPWV can be used to monitor the response of arterial stiffness to therapy or its progression^10,11^. Moreover, the baPWV has become a standard for characterizing vascular aging because arterial stiffness captures the total damage experienced by the arterial wall and provides a more holistic perspective than traditional metrics such as blood pressure or lipid levels^1,12,13^.

In the present study, vascular aging categorized based on arterial stiffness was associated with unfavorable outcomes after acute ischemic stroke. The concept of vascular aging can be used to express cardiovascular risk based on estimated biological age and for prognosticating its occurrence in the general population^4,5^. Vascular aging can vary in presentation, independent of risk factors, due to imbalances in the protective genetic/molecular pathways^3^. Intrinsic vulnerability to vascular aging, such as EVA, may exacerbate arterial stiffness and future vascular events. Conversely, resistance to vascular aging, such as SUPERNOVA, may increase resilience to vascular disease^1^. Therefore, vascular aging phenotyping may provide additional information to predict stroke prognosis.

The EVA phenotype is characterized by abnormally high arterial stiffness compared with other phenotypes. Increasing evidence indicates the deleterious effect of stiffer arteries^1^. In several studies, arterial stiffness was shown more accurate predictor of cardiovascular events than conventional risk scores^13,14^. Meta-analysis showed an increase of 1 m/s in baPWV was associated with a 12% increase in total cardiovascular events, a 13% increase in cardiovascular mortality, and a 6% increase in all-cause mortality^13^. In patients with stable angina, arterial stiffness was associated with all‐cause mortality in addition to traditional risk factors^14^. Similarly, in patients with acute ischemic stroke, arterial stiffness was associated with short-term outcomes such as worsening of neurological symptoms and a slower early recovery^15,16^. Arterial stiffness was also linked to worse long-term outcomes in terms of all-cause and vascular mortality after acute ischemic stroke^17^. Additionally, we found that EVA patients had a higher prevalence of hypertension, diabetes, and atrial fibrillation compared to NVA patients, which is consistent with previous research of arterial stiffness^9,18^. Therefore, increased arterial stiffness may be a contributing factor to the correlation between EVA and adverse outcomes in patients with acute ischemic stroke.

In our study, the deteriorating effect of EVA on stroke outcome was more obvious in certain subgroups. In patients with diabetes, EVA showed a higher prognostic value of poor functional outcome than in subjects without. This result was supported by previous studies that showed diabetes accelerates vascular aging and impairs functional recovery after stroke^19,20^. Patients with diabetes also tended to have more risk factors such as hypertension and dyslipidemia (Supplementary Table 1). Moreover, EVA had a greater effect on all-cause mortality in patients with atrial fibrillation than in subjects without. The supplemental finding showed patients with atrial fibrillation had higher CHA_2_DS_2_-VASc scores, were older, and had a history of previous stroke (Supplementary Table 2). Therefore, the burden of these risk factors may have influenced the results^21^. However, the prominent effect of EVA was not consistent across both outcomes. Further studies are needed to elucidate the interplay between diabetes, atrial fibrillation, and vascular aging.

In the current study, EVA was associated with poor functional outcome and mortality, albeit in small numbers. Previous studies included relatively large numbers of EVA subjects, but these were community-based and general population studies^4,5,9^. The researchers also used the extreme distributions of upper and lower 10% to define EVA and SUPERNOVA, respectively^4,5^. This definition could create a gray zone where some NVA subjects are included with EVA and SUPERNOVA subjects^1^. In addition, only EVA was addressed in stroke studies and correlations with cardiovascular risk factors in young or low-risk stroke subtypes were reported^22,23^. However, the present study used an upper and lower bound of 2.5% for vascular aging phenotyping and included acute ischemic stroke patients, which is a strength of the study. SUPERNOVA implies extremely low arterial stiffness for chronological age; however, in this study, SUPERNOVA was not found to be superior to NVA in terms of prognosis. A potential explanation is the higher prevalence of atrial fibrillation and other cardioembolism in SUPERNOVA patients (Table 1), which may mitigate the positive prognostic effect.

This study had several limitations. First, relatively mild stroke patients were included because baPWV measurements require patient cooperation. Second, instead of the gold standard carotid-femoral PWV, baPWV was used for vascular aging phenotyping. However, a strong correlation between the two PWV modalities was reported in previous research^24^. Third, the study was structured as an observational and retrospective analysis, but patients were sequentially enrolled and underwent the same clinical treatment protocol. Fourth, baPWV is related to vascular aging but not identical to it. Therefore, our results show a possible link between vascular aging phenotypes and stroke prognosis.

## Conclusion

In the present study, the EVA phenotype was an independent prognostic factor predicting short-term poor functional outcomes and long-term all-cause mortality in acute ischemic stroke patients undergoing baPWV. This finding indicates the vascular aging phenotype, particularly EVA, could help identify high-risk patients among those with relatively mild stroke.

## Methods

### Study population

Patients diagnosed with acute ischemic stroke within 7 days of onset from January 2012 to December 2018 were consecutively enrolled. Every patient underwent neuroimaging with either magnetic resonance imaging (MRI) and/or computed tomography (CT). The condition of the cerebral vessels was assessed using cerebral angiography with MR angiography, CT angiography, or digital subtraction angiography. Systemic evaluations included chest radiography, 12-lead electrocardiography, routine blood tests, and lipid profiling. Transthoracic echocardiography, transesophageal echocardiography, or cardiac CT were performed for selected patients. As part of the standard evaluation, baPWV was measured in all patients, except for subjects with decreased consciousness, impending brain herniation, poor systemic conditions, or lack of informed consent. The median interval between stroke onset and baPWV measurement was 4 days (interquartile range, 2–5 days). The stroke subtype was determined using the Trial of ORG 10172 in Acute Stroke Treatment classification^25^. Patients were appropriately managed and treated according to the guidelines for acute ischemic stroke^26^.

### Assessment and vascular aging phenotype

Vascular aging was assessed using baPWV (VP-1000 Plus; Colin Co., Ltd., Komaki, Japan), which is the distance between the brachial and the ankle divided by the delay time between the initial point of the brachial pulse wave and the ankle pulse wave. Vascular aging phenotype was taken from a previous study that used the age quintile-specific 2.5th and 97.5th percentile of baPWV as cutoffs for defining SUPERNOVA and EVA, respectively^9^. Briefly, a baPWV value below the age quintile-specific 2.5th percentile was defined as SUPERNOVA, a baPWV value above the age quintile-specific 97.5th percentile as EVA, and the remaining patients were considered NVA (Supplementary Fig. 2).

### Follow-up and outcomes

Patients were followed in the outpatient clinic or with a structured telephone interview at 3 months and annually after discharge. Poor functional outcome was defined as the modified Rankin Scale score ≥ 3 at 3 months. MACE was defined as any development of stroke recurrence, acute coronary syndrome, or all-cause mortality. Stroke recurrence refers to newly developed neurologic symptoms relevant to lesions on brain imaging. The censoring date was December 31, 2019. If a patient's final visit occurred prior to this date, that last visit's date was used as the censoring date. The Institutional Review Board of the Severance Hospital of the Yonsei University Health System approved this study, and due to the retrospective nature of the study, the need for informed consent was waived (approval number: 4-2023-0974). This study was conducted ethically in accordance with the World Medical Association Declaration of Helsinki.

### Statistical analysis

Categorical variables are presented as counts with percentages. Continuous variables are presented as either the mean with standard deviation or the median with interquartile range. The significant intergroup difference was assessed using the chi-square and Fisher’s exact tests for categorical variables or the one-way analysis of variance and the Kruskal–Wallis tests for continuous variables. Logistic regression analysis was performed to observe the significant factors for poor functional outcome. Survival curves were plotted using the Kaplan–Meier analysis and compared with the log-rank test. Cox proportional hazards regression analysis was performed to determine the significant factors associated with long-term outcomes. To identify the independent association of vascular aging with outcomes, multivariable analysis was utilized with adjustment for covariates such as age, sex, body mass index, NIHSS score at admission, and variables that were significant (p < 0.05) in univariable analysis. For the covariate stroke subtype, small vessel occlusion was used as a reference because it had a better prognosis compared to the other subtypes (Supplementary Table 4). The independent association between vascular aging and long-term outcomes was further confirmed by adjusting for the ASCVD risk factors such as age, sex, hypertension, diabetes, current smoking, total cholesterol, and high-density lipoprotein cholesterol. Subgroup analysis was performed using multivariable logistic or Cox regression analyses based on patient characteristics. All statistical analyses were conducted with SPSS 26 (Chicago, IL, USA) and two-tailed p < 0.05 was considered statistically significant.

### Supplementary Information

Supplementary Figures.

Supplementary Tables.

## Data Availability

The study data are available from the corresponding author upon reasonable request and with the permission of all contributing authors.
