# Supplementary material for: Early vascular aging determined by brachial-ankle pulse wave velocity and its impact on ischemic stroke outcome: a retrospective observational study
Source: Sci Rep. 2024 Jun 13;14:13659. doi: 10.1038/s41598-024-62847-w (PMC11176412; doi:10.1038/s41598-024-62847-w)
Supplement: Supplementary file 1 — Supplementary Figures. [file 41598_2024_62847_MOESM1_ESM.docx]

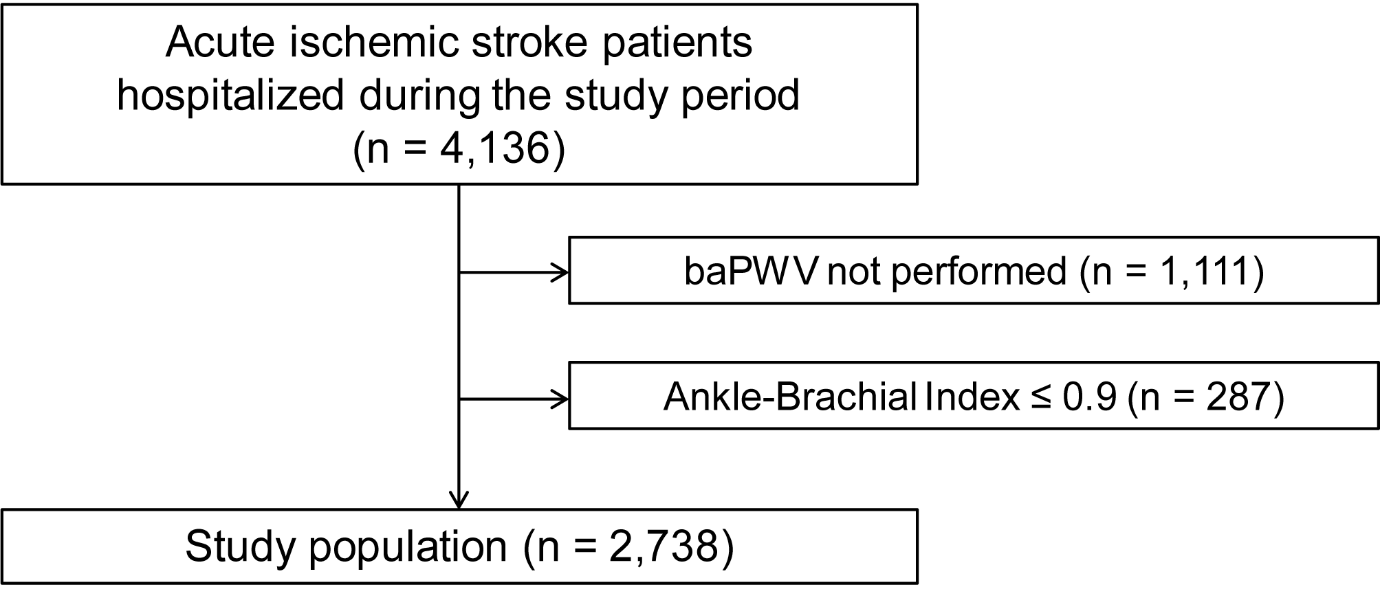


**Supplementary Figure 1. Study population flow chart.** baPWV, brachial-ankle pulse wave velocity.

**
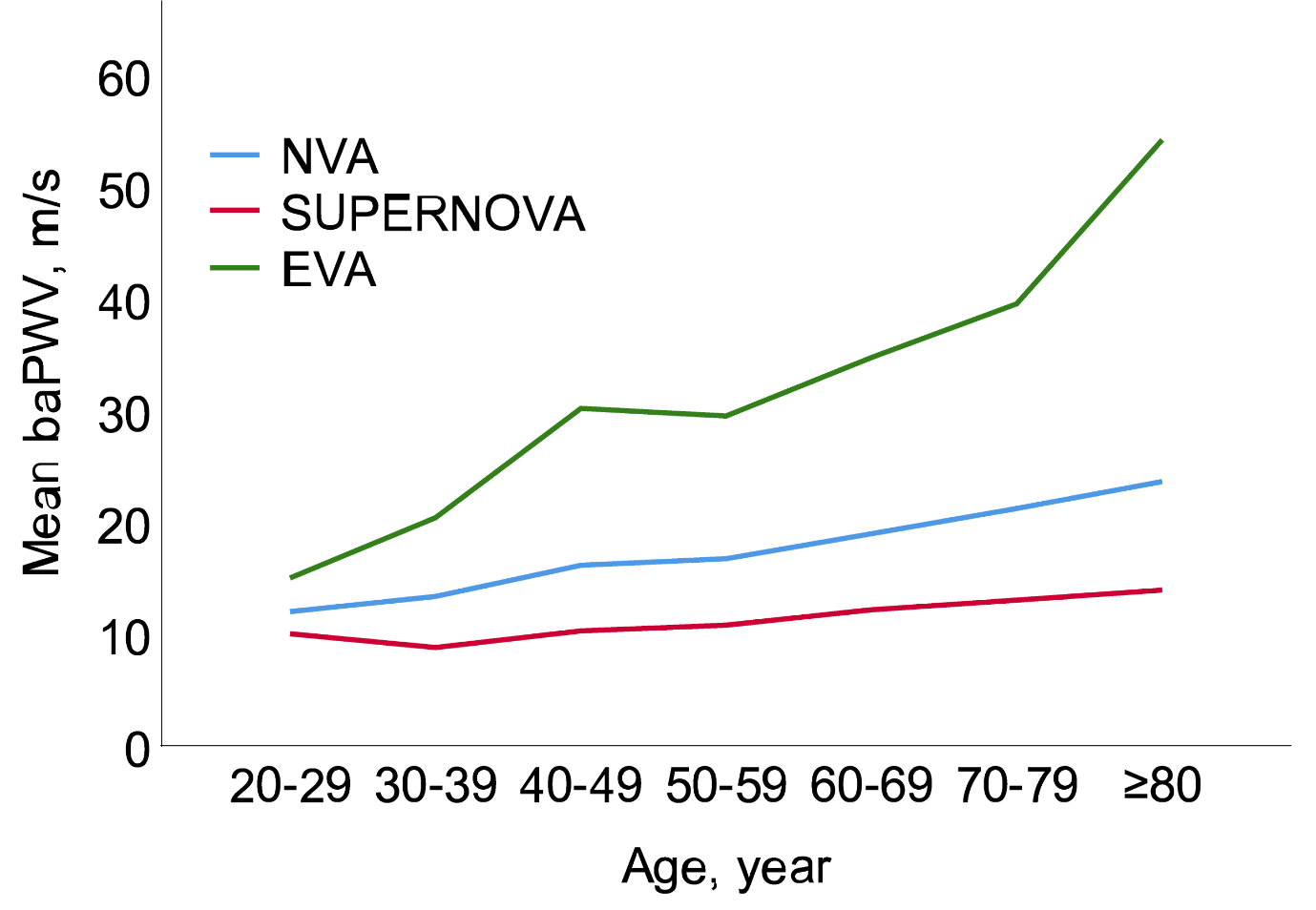
**

**Supplementary Figure 2. Mean values of baPWV by age and vascular aging.** baPWV, brachial-ankle pulse wave velocity. EVA, early vascular aging; NVA, normal vascular aging; SUPERNOVA, supernormal vascular aging.
