# Supplementary material for: Early vascular aging determined by brachial-ankle pulse wave velocity and its impact on ischemic stroke outcome: a retrospective observational study
Source: Sci Rep. 2024 Jun 13;14:13659. doi: 10.1038/s41598-024-62847-w (PMC11176412; doi:10.1038/s41598-024-62847-w)
Supplement: Supplementary file 2 — Supplementary Tables. [file 41598_2024_62847_MOESM2_ESM.docx]

# Supplementary Table 1. Univariable Cox regression analysis of long-term outcomes

|  | MACE |  | Stroke recurrence |  | All-cause mortality |  |
| --- | --- | --- | --- | --- | --- | --- |
|  | HR (95% CI) | *p-*value | HR (95% CI) | *p-*value | HR (95% CI) | *p-*value |
| Age, y | 1.037 (1.029‒1.045) | <0.001 | 1.016 (1.006‒1.026) | 0.002 | 1.082 (1.069‒1.096) | <0.001 |
| Sex(male) | 1.160 (0.966‒1.392) | 0.112 | 1.168 (0.906‒1.505) | 0.231 | 1.081 (0.828‒1.412) | 0.565 |
| Body mass index, kg/m^2^ | 0.918 (0.892‒0.945) | <0.001 | 0.980 (0.944‒1.018) | 0.301 | 0.852 (0.815‒0.890) | <0.001 |
| NIHSS at admission | 1.050 (1.033‒1.066) | <0.001 | 1.015 (0.989‒1.041) | 0.269 | 1.097 (1.077‒1.118) | <0.001 |
| Hypertension | 1.281 (1.038‒1.581) | 0.021 | 1.521 (1.120‒2.067) | 0.007 | 1.105 (0.819‒1.491) | 0.514 |
| Diabetes | 1.568 (1.310‒1.877) | <0.001 | 1.423 (1.107‒1.830) | 0.006 | 1.592 (1.223‒2.071) | 0.001 |
| Dyslipidemia | 0.953 (0.757‒1.200) | 0.681 | 1.112 (0.820‒1.508) | 0.495 | 0.668 (0.455‒0.980) | 0.039 |
| Current smoking | 0.724 (0.574‒0.913) | 0.006 | 0.764 (0.557‒1.049) | 0.096 | 0.512 (0.349‒0.752) | 0.001 |
| Atrial fibrillation | 1.561 (1.283‒1.900) | <0.001 | 1.345 (1.014‒1.784) | 0.040 | 2.328 (1.778‒3.048) | <0.001 |
| Coronary artery disease | 1.269 (1.056‒1.527) | 0.011 | 1.155 (0.892‒1.495) | 0.276 | 1.052 (0.796‒1.390) | 0.721 |
| Previous stroke | 1.538 (1.248‒1.895) | <0.001 | 1.629 (1.224‒2.167) | 0.001 | 1.763 (1.311‒2.371) | <0.001 |
| Total cholesterol, mg/dL | 0.994 (0.992‒0.996) | <0.001 | 0.997 (0.994‒1.000) | 0.054 | 0.991 (0.987‒0.994) | <0.001 |
| HDL-C, mg/dL | 0.990 (0.982‒0.998) | 0.014 | 0.995 (0.984‒1.006) | 0.376 | 0.988 (0.976‒1.000) | 0.045 |
| LDL-C, mg/dL | 0.993 (0.991‒0.996) | <0.001 | 0.997 (0.993‒1.000) | 0.068 | 0.989 (0.985‒0.993) | <0.001 |
| Triglyceride, mg/dL | 0.999 (0.997‒1.000) | 0.027 | 1.000 (0.998‒1.001) | 0.507 | 0.996 (0.994‒0.999) | 0.001 |
| Stroke subtypes |  |  |  |  |  |  |
| Small vessel occlusion | Reference |  | Reference |  | Reference |  |
| Large artery atherosclerosis | 2.035 (1.342‒3.084) | 0.001 | 1.891 (1.111‒3.221) | 0.019 | 2.549 (1.180‒5.507) | 0.017 |
| Cardioembolism | 1.894 (1.273‒2.818) | 0.002 | 1.506 (0.900‒2.520) | 0.119 | 3.805 (1.842‒7.861) | <0.001 |
| Undetermined causes | 1.785 (1.212‒2.628) | 0.003 | 1.432 (0.870‒2.358) | 0.158 | 3.066 (1.494‒6.294) | <0.002 |
| **Vascular aging** |  |  |  |  |  |  |
| NVA | Reference |  | Reference |  | Reference |  |
| SUPERNOVA | 1.063 (0.613‒1.845) | 0.828 | 0.744 (0.307‒1.803) | 0.512 | 1.048 (0.465‒2.358) | 0.911 |
| EVA | 1.672 (1.030‒2.713) | 0.038 | 0.710 (0.265‒1.908) | 0.498 | 2.557 (1.429‒4.576) | 0.002 |

CI, confidence interval; EVA, early vascular ageing; HDL-C, high-density lipoprotein cholesterol; HR, hazard ratio; LDL-C, low-density lipoprotein cholesterol; NIHSS, National Institutes of Health Stroke Scale; NVA, normal vascular ageing; SUPERNOVA, supernormal vascular ageing.

**Supplementary Table 2. Demographic and clinical characteristics in patient with EVA**

|  | Diabetes |  | *p*-value |
| --- | --- | --- | --- |
|  | Yes (n=36) | No (n=31) |  |
| Age, y | 65.56±14.00 | 71.55±14.09 | 0.086 |
| Body mass index, kg/m^2^ | 23.60±2.48 | 22.71±2.84 | 0.176 |
| NIHSS score at admission | 3.0 [2.0, 4.0] | 2.0 [1.0, 7.0] | 0.489 |
| Hypertension | 34 (94.4) | 23 (74.2) | 0.036 |
| Dyslipidemia | 11 (30.6) | 3 (9.7) | 0.036 |
| Current smoking | 5 (13.9) | 5 (16.1) | 1.000 |
| Atrial fibrillation | 12 (33.3) | 12 (38.7) | 0.647 |
| Coronary artery disease | 14 (38.9) | 9 (29.0) | 0.397 |
| Previous stroke | 10 (27.8) | 5 (16.1) | 0.254 |

P-value was obtained with the independent two-sample t-test and Mann-Whitney U tests for continuous variables or the Chi-square and Fisher’s exact tests for categorical variables. NIHSS, National Institutes of Health Stroke Scale; EVA, early vascular aging.

**Supplementary Table 3. Demographic and clinical characteristics in patient with EVA**

|  | Atrial fibrillation |  | *p*-value |
| --- | --- | --- | --- |
|  | Yes (n=24) | No (n=43) |  |
| Age 65-74 | 5 (20.8) | 10 (23.3) | 0.820 |
| Age ≥75 | 16 (66.7) | 10 (23.3) | <0.001 |
| Sex(female) | 11 (45.8) | 23 (53.5) | 0.548 |
| Hypertension | 19 (79.2) | 38 (88.4) | 0.476 |
| Diabetes | 12 (50.0) | 24 (55.8) | 0.647 |
| Coronary artery disease | 9 (37.5) | 14 (32.6) | 0.683 |
| Previous stroke | 9 (37.5) | 6 (14.0) | 0.027 |
| CHA_2_DS_2_-VASc score | 4.0 [3.0, 6.0] | 3.0 [2.0, 4.0] | 0.007 |

The CHA_2_DS_2_-VASc score was calculated as a total by assigning 1 point for age 65-74, female, hypertension, diabetes, and coronary artery disease, and 2 points for age 75 and older and previous stroke. P-values were obtained with the Chi-square and Fisher’s exact tests for categorical variables or the Mann-Whitney U test for a continuous variable. EVA, early vascular aging.

**Supplementary Table 4. Cox regression analysis of major adverse cardiovascular event**

|  | Univariable (pairwise comparison) | | |  |  |  |
| --- | --- | --- | --- | --- | --- | --- |
|  | HR (95% CI) | p-value | HR (95% CI) | p-value | HR (95% CI) | p-value |
| Stroke subtype |  |  |  |  |  |  |
| SVO | Reference |  |  |  |  |  |
| LAA | 2.035 (1.342‒3.084) | 0.001 | Reference |  |  |  |
| CE | 1.894 (1.273‒2.818) | 0.002 | 0.931 (0.720‒1.203) | 0.584 | Reference |  |
| UC | 1.785 (1.212‒2.628) | 0.003 | 0.877 (0.689‒1.116) | 0.286 | 0.942 (0.766‒1.159) | 0.573 |
|  | Multivariable (pairwise comparison)^a^ | | |  |  |  |
|  | HR (95% CI) | p-value | HR (95% CI) | p-value | HR (95% CI) | p-value |
| Stroke subtype |  |  |  |  |  |  |
| SVO | Reference |  |  |  |  |  |
| LAA | 1.824 (1.203‒2.768) | 0.005 | Reference |  |  |  |
| CE | 1.738 (1.165‒2.592) | 0.007 | 0.952 (0.734‒1.236) | 0.713 | Reference |  |
| UC | 1.700 (1.153‒2.508) | 0.007 | 0.932 (0.731‒1.188) | 0.569 | 0.979 (0.793‒1.207) | 0.566 |

CE, cardioembolism; CI, confidence interval; HR, hazard ratio; LAA, large artery atherosclerosis; SVO, small vessel occlusion; UC, undetermined causes.

^a^adjusted for the atherosclerotic cardiovascular disease risk factors (age, sex, hypertension, diabetes, current smoking, total cholesterol, and high-density lipoprotein cholesterol).
